# Computational analysis and expression profiling of potassium transport-related gene families in mango (*Mangifera indica*) indicate their role in stress response and fruit development

**DOI:** 10.3389/fpls.2022.1102201

**Published:** 2023-01-23

**Authors:** Lin Tan, Muhammad Waqas, Abdul Rehman, Muhammad Abdul Rehman Rashid, Sajid Fiaz, Hamid Manzoor, Farrukh Azeem

**Affiliations:** ^1^ Haikou Experimental Station, Chinese Academy of Tropical Agricultural Sciences, Haikou, Hainan, China; ^2^ Department of Bioinformatics and Biotechnology, Government College University, Faisalabad, Pakistan; ^3^ Department of Plant Breeding and Genetics, The University of Haripur, Haripur, Pakistan; ^4^ Institute of Molecular Biology and Biotechnology, Bahauddin Zakariya University, Multan, Pakistan

**Keywords:** mango, potassium, channel, transporter, fruit development, drought stress

## Abstract

Mango (*Mangifera indica*) fruit is known for its taste, health benefits, and drought tolerance. Potassium (K^+^) is one of the most abundant ions in a plant cell. It is important for various biological functions related to plant growth, development, and flowering/fruiting. It significantly contributes to fruit yield, quality, and drought tolerance in plants. However, molecular mechanisms comprising K^+^ transport in mango are least known. In the present study, 37 members of K^+^ transport-related genes (PTGs) were identified in mango, which include 22 K^+^ transporters (16 HAKs, 1 HKT, and 6 KEAs) and 15 K^+^ channels (6 TPKs and 8 Shakers). All PTGs were predicted to be expressed at the plasma membrane and possess characteristic motifs and domains. Phylogenetic analysis identified a strong kinship of PTGs among *Oryza sativa*, *Arabidopsis thaliana*, *Cicer arietinum*, *Malus domestica*, and *M. indica*. The promoter analysis identified 60 types of *cis*-elements related to various biological processes. RNA-seq-based expression profiling identified that *MiTPK1.2*, *MiHAK1*, *MiHAK2.1*, *HAK6.1*, and *MiAKT1.1* were most upregulated in roots and that *MiKEA2*, *MiAKT2*, and *MiAKT1* were upregulated in leaves. Moreover, *MiAKT6*, *MiHAK1.1*, *MiKAT2*, *MiKAT2.1*, *MiHKT1*, *MiTPK1.1*, *MiHAK7*, and *MiHAK12* were highly expressed during the five growth stages of mango fruit. The current study is the first comprehensive report on K^+^ transport system in tropical fruits. Therefore, it will provide the foundation knowledge for the functional characterization of K^+^ genes in mango and related plants.

## 1 Introduction

Mango (*Mangifera indica*) is a widespread, evergreen, and one of the most dominant tropical fruits worldwide, being the sixth most cultivated fruit after bananas (*Musa acuminata*), watermelons (*Citrullus lanatus*), apples (*Malus domestica*), oranges (*Citrus sinensis*), and grapes (*Vitis vinifera*) (Ngamchuachit et al., 2015). Its bright color, distinctive quality, unique taste, and nutritional value have promoted its consumption. The mango fruit trade continues to grow and develop in the food service and dime markets (Celestin, 2019). Mango can be used as a pudding, fresh juice, and extracted products; refined into jam; or used as a jelly bean. With extensive cultivation of this fruit, India is known as a major producer with 25 million tonnes in 2020 (FAOSTAT, 2022), as compared to other countries like Pakistan, China, Indonesia, Malawi, and Mexico (Galán Saúco, 2002). Mango trees can withstand drought conditions, but these may affect the overall quality of the fruit (Cleveland, 2012). Since peak fruit development occurs in the dry season, water requirement is critical (Singh and Kushwaha, 2006; Snowden et al., 2014). The standard ranking of export mangoes (flavor, size, shape, and color) can contribute an additional 30%–50% to the payout (Laurio, 2021). Farmers are encouraged to increase the irrigation for this crop because of its tremendous impact on profitable yield. However, water scarcity and the enormous expenditure of energy required to raise irrigation water during the peak season have threatened fruit production and cultivation (Zikki, 2020). For this reason, solid agronomic and irrigation practices are applied at the farm level to help plants survive drought stress. However, investigations into mango drought stress tolerance are relatively scarce.

K^+^ acquisition is one of the most important issues covering organic agriculture, and it is because most organic sources of K^+^ are poorly soluble, limiting plant growth (Council, 1993). Due to its role in protein synthesis, ionic stability, photosynthesis, stress tolerance, translocation of photosynthates, and the initiation of several plant enzymes, K^+^ is an essential component for plant maturation and final production (Hossain et al., 2021; Ul Hassan et al., 2021). K^+^ plays a unique role in the generation of starch, blooming, and fruit output (Farooq et al., 2014). However, increased K^+^ rates in plants can result in an imbalance in magnesium and calcium nutrition (Nahar et al., 2015). For plant growth, the main sources are the utilization of chemical K^+^ fertilizer and the disintegration of K^+^-minerals (Etesami et al., 2017; Bahadur et al., 2019). The application of appropriate K^+^ fertilizer to mango trees increases fruit yield and value. Nevertheless, unconstrained fertilizer usage can result in financial loss to farmers, and excessive use of non-renewable resources is raising concerns about large-scale sustainable development (Bahadur et al., 2019; Wang et al., 2022). Foliar application of K^+^ is also practiced to improve fruit yield. Therefore, to amend the K^+^ utilization for mango, there is a need to acknowledge K^+^ transport mechanisms.

Different proteins in plants that control cellular K^+^ absorption and distribution include both channels and transporters. Voltage-dependent channel proteins mediating K^+^ transport include shaker-like channels, voltage-independent tandem-pore K^+^ (TPK) channels, and two-pore channels (TPCs) (Voelker et al., 2010). Five subgroups are used to classify the Shaker family: weak inward rectifying channels, inward rectifying channels with KAT-like characteristics, outward rectifying channels, inward rectifying channels with AKT-like characteristics, and the silent/regulatory subunit (González et al., 2015). Moreover, the carrier-like KUP/HAK/KT family (Azeem et al., 2022), HKT uniporters and symporters (Sahi et al., 2022), and KEA antiporters (Wang et al., 2017) also manifested their involvement in this mechanism. The KUP/HAK/KT family may regulate K^+^ with great affinity (Ye et al., 2013). The assimilation and homeostasis of K^+^ and sodium ions depend on the HKT proteins. In plants, there are two kinds of HKTs, namely, HKT1-like and HKT 2-like. The Na^+^ uniporters make up class I HKTs, whereas Na^+^ and K^+^ symporters make up class II HKTs (Riedelsberger et al., 2021). The KEA proteins are probably similar to bacterial KefC K^+^/H^+^ antiporters (Waters et al., 2013; Assaha et al., 2015). Most KEAs are shown to control the pH value of stroma and thylakoids at the chloroplast membrane (Sun et al., 2018). In endomembrane cells, KEA 4, 5, and 6 help to maintain pH and K^+^ homeostasis in a balanced manner (Sze and Chanroj, 2018; Zhu et al., 2018). Pore domains (PDs) are used to calculate the K^+^ channels, which are heterodimeric proteins with trans-membrane sections. Functional multimeric proteins are associated with four PDs to make a navigation pathway of channels. P domain of the K^+^ channels contains a highly conserved motif, i.e., “GYGD/E”. In *Arabidopsis thaliana*, 15 K^+^-selective channels, including one K^+^ inward rectifier (Kir-like), five tandem-pore K^+^ channels (TPK), and nine voltage-gated ion channels, are grouped into three families based on their physiography. Additionally, K^+^ transporters are divided into three families—the Trk/HKT family of high-affinity K^+^ transporters, the KEA (K^+^/H^+^ antiprotons) family of K^+^ efflux antiporters, and the KUP/HAK/KT family of K^+^ uptake permeases—which collectively have 13 members (1 member) (Aranda-Sicilia et al., 2012).

The current study was designed to identify key protein families involved in K^+^ transport. For this purpose, a combination of bioinformatics approaches was used to characterize potential PTGs, and expression profiling was conducted using next-generation sequencing (NGS) data available at the National Center for Biotechnology Information (NCBI).

## 2 Material and methods

### 2.1 Identification and sequence analysis of K^+^ transport-related gene families in *M indica*

For the identification of PTG families in *M. indica*, PTG sequences from *A. thaliana*, *Cicer arietinum*, and *Cajanus cajan* were used to perform a BLAST search in the NCBI (https://www.ncbi.nlm.nih.gov) and Phytozome (https://phytozome-next.jgi.doe.gov/) databases (Johnson et al., 2008; Goodstein et al., 2012). An *E*-value 1e^−10^ was used as the cutoff value BLAST search. After retrieving all the sequencing, we utilized SMART and pfam tools to verify the accuracy of K^+^ transporting gene family in *M. indica* and deleted the sequences that lacked the conserved domains. To enhance the accuracy of our analysis, we deleted genes having common start positions in the genome representing the same locus or splice variants.

### 2.2 Multiple sequence analysis and phylogenetic analysis of K^+^ transporting gene family

The MEGA7 software with default parameters was used for multiple sequence alignment of PTG sequences of five different species, i.e., *A. thaliana*, *M. domestica*, *Oryza sativa*, *C. arietinum*, and *M. indica*. Moving forward after multiple sequence alignment (MSA) of 198 protein sequences of the PTG families, with the use of the IQ TREE web server (http://iqtree.cibiv.univie.ac.at/), a phylogenetic tree was built based upon the maximum likelihood method. The tree was designed and visualized by using a web-based tool ITOL (https://itol.embl.de/) (Schultz et al., 2000).

### 2.3 Physiochemical properties of K^+^ transporting gene family members in *M. indica*

The physicochemical properties of PTG family members were predicted by using a web-based tool ProtParam (https://web.expasy.org/protparam/) with default options. Gene ID, chromosomal locations, number of nucleotides in genomic DNA and mRNA, and number of exons were identified by using the NCBI gene database (https://www.ncbi.nlm.nih.gov/gene). The subcellular localization of K^+^ transporting proteins was predicted by using a web-based tool ProtCamp 9.0 (ProtComp—Predict the sub-cellular localization for Plant proteins (softberry.com), and then, these locations were also verified by another online tool, CELLO v.2.5 (http://cello.life.nctu.edu.tw/), by using protein sequences of K^+^ transporting gene family (Garg et al., 2016; Mehanny et al., 2022).

### 2.4 Sequence analysis of PTG family members in *M. indica*

To predict the exons and introns of the K^+^ transporting genes, genomic DNA and cDNA sequences of all the members of K^+^ transporting genes of *M. indica* were retrieved from the NCBI database in FASTA format. Exons and introns in these genes were predicted using an online tool named Gene Structure Display Server (GSDS) 2.0 (http://gsds.gao-lab.org/). Motifs of the K^+^ transporting gene family were identified by an online software called MEME (https://meme-suite.org/meme/tools/meme). To predict the conserved motifs, the following parameters were selected: one occurrence per sequence (oops), number of motifs of 10, motifs with at least a width of 10, and utmost width of 50. Motif logos were also made by MEME (Guo et al., 2007; Bailey et al., 2009).

### 2.5 Domain analysis of K^+^ transporting genes in *M. indica*

To predict the conserved domains of the K^+^ transporting gene family in *M. indica*, the NCBI-CDD database (https://www.ncbi.nlm.nih.gov/Structure/cdd/wrpsb.cgi) was used in which all the sequences were searched against the Pfam database. These results were also verified by the SMART tool (http://smart.embl-heidelberg.de/). Then, conserved domains of the K^+^ transporting gene family were visualized by the TB tool, using the results of the Pfam database.

### 2.6 *Cis*-regulatory elements of K^+^ transporting gene family in *M. indica*

For determining, the *cis*-acting regulatory elements of the K^+^ transporting genes in *M. indica*, an approximately 2,000-bp promoter region was retrieved for each gene from the NCBI gene page. After the promoter sequence was retrieved, the PlantCare database (http://bioinformatics.psb.ugent.be/webtools/plantcare/html/) was employed to predict the *cis*-acting regulatory elements in promoter regions of K^+^ transporting genes. The PlantCare results were downloaded and opened in the Excel sheets that were used to anticipate the *cis*-acting regulatory elements in TBtools software (Lescot et al., 2002).

### 2.7 Chromosomal distribution of K^+^ transporting genes

The gene location (initial and terminating position) of K^+^ transporting genes present on the chromosomes was identified by the NCBI gene database. The chromosome number for each member of the PTG family was also identified from the NCBI database. The chromosomal location was visualized by a Ritchie lab tool, Phenograms (http://visualization.ritchielab.org/phenograms/plot).

### 2.8 Gene expression profile of K^+^ transporting genes using NGS data and qRT-PCR

For evaluation of the gene expression configuration of the K^+^ transporting genes under abiotic stress (drought stress with bio-project), genome and gff files were downloaded from the NCBI-SRA [Home—SRA—NCBI (nih.gov)] database after the genome, and gff files were retrieved from the NCBI-genome database. The galaxy server [Galaxy (usegalaxy.org)] was utilized to obtain the fragments per kilobase of transcript per million mapped reads (FPKM) values, and the expression pattern was shown as a heat map using TB tools. Drought stress was applied to 1-year-old mango plants (Chaunsa) grown in soil-filled pots (one plant/pot) under an ambient environment (12-h photoperiod, 50%–60% relative humidity, 25°C ± 1°C day/night temperature). Drought stress was imposed by watering each pot (n = 6) with 1 L of 200 g/L PEG-6000. Leaves were collected 2, 4, and 7 days after the start of treatment. To validate the expression profiles of NGS data, real-time RT-qPCR was used. For this purpose, RNA was extracted from mango leaves using the TRIzol reagent and was quantified using NanoDrop 2000 (Thermo Fisher Scientific, Waltham, MA, USA). With the use of 1 µg of RNA and the Maxima H-minus First-Strand cDNA synthesis kit, the RNA was reverse transcribed, and cDNA was stored at −20°C. An iTaq Universal SYBR Green Super-Mix and a qRT-PCR detection equipment (CFX96 Touch RT PCR Detection System, Bio-Rad Labs, Hercules, CA, USA) were used to perform the qRT-PCR. Gene-specific primers were mapped using an online program “Oligo Calculator” at mcb.berkeley.edu/labs/krantz/tools/oligocalc.html (accessed 26 July 2022), and the specificity of primers was verified using NCBI-primer BLAST algorithm (https://www.ncbi.nlm.nih.gov/tools/primer-blast/) (retrieved on 26 July 2022). Actin (LOC123192663) was used as a reference gene (Yao et al., 2022). The 2^−ΔΔCT^ method is used to calculate respective gene expression levels on the basis of three biological replicates (Livak and Schmittgen, 2001).

## 3 Results

### 3.1 Computational identification and characterization of K^+^ transport-related genes

A total of 37 PTGs were identified in *M. indica* genome (**Table 1**). Physiochemical characteristics of K^+^ transporter in *M. indica*, such as the total number of amino acids (aa), molecular weights (MW), aliphatic index, gravy, and hypothetical isoelectric points (pI), were identified using the web-based tool Expasy-protparam. Subcellular localization was also identified using cello 2.0. The length of protein ranged from 340aa to 1208aa. Molecular weight varied from 39.05 to 130.77 kDa, and the pI values start from 4.92 to 9.47 in which 16 of the proteins were acidic and 20 were basic. Interestingly, the MiHAK1.1 protein possessed a pI value of 7.01. GRAVY values of AKT and KAT-like proteins were negative (hydrophilic), while the rest of the proteins were hydrophobic, and subcellular localization showed that the location of these proteins is in the plasma membrane (**Table 1**).

**Table 1:** Physiochemical properties of K^+^ transport-related genes in *Mangifera indica*.

Sr. no.	Accession no.	Gene names	Gene ID	Chr. No.	Exon	Protein length (aa)	Molecular weight (Da)	pI	Gravy	Aliphatic index	Subcellular localization
**1**	XP_044508080.1	*MiHKT1*	LOC123227371	10	3	498	56,848.8	9.47	0.298	104	Plasma membrane
**2**	XP_044473757.1	*MiHAK1.1*	LOC123202089	2	9	753	83,293.7	7.01	0.489	111	Plasma membrane
**3**	XP_044493735.1	*MiHAK6.1*	LOC123217052	5	8	781	87,042	8.59	0.336	108.92	Plasma membrane
**4**	XP_044466396.1	*MiHAK6*	LOC123196431	14	8	776	86,449.4	8.15	0.377	109.11	Plasma membrane
**5**	XP_044508633.1	*MiHAK2.1*	LOC123227631	10	12	793	88,856.8	6.92	0.375	108.15	Plasma membrane
**6**	XP_044509128.1	*MiHAK10*	LOC123228011	10	8	794	88,971.3	8.53	0.366	109.58	Plasma membrane
**7**	XP_044492982.1	*MiHAK12*	LOC123216588	5	9	837	93,080	5.78	0.373	107.35	Plasma membrane
**8**	XP_044490120.1	*MiHAK7*	LOC123214428	4	10	848	94,175.8	5.66	0.314	105.61	Plasma membrane
**9**	XP_044479071.1	*MiHAK8.1*	LOC123206042	Unknown	9	775	86,717.8	7.53	0.389	110.76	Plasma membrane
**10**	XP_044465942.1	*MiHAK4*	LOC123196123	14	9	784	87,470.5	8.58	0.448	111.39	Plasma membrane
**11**	XP_044466397.1	*MiHAK8*	LOC123196431	14	8	775	86,321.2	8.15	8.15	109.25	Plasma membrane
**12**	XP_044512380.1	*MiHAK5*	LOC123230292	12	7	804	89,457.1	8.57	0.301	108.93	Plasma membrane
**13**	XP_044501404.1	*MiHAK5.3*	LOC123222612	8	8	779	87,506.1	8.04	0.221	105.56	Plasma membrane
**14**	XP_044502490.1	*MiHAK5.1*	LOC123223391	8	8	772	86,120.4	6.72	0.279	106	Plasma membrane
**15**	XP_044492757.1	*MiHAK5.2*	LOC123216406	5	8	780	87,931.8	7.54	0.233	104.79	Plasma membrane
**16**	XP_044467837.1	*MiHAK1*	LOC123197584	15	10	737	81,919.4	8.68	0.522	112.55	Plasma membrane
**17**	XP_044461170.1	*MiHAK2*	LOC123192615	12	11	793	88738.3	6.54	0.358	107.53	Plasma membrane
**18**	XP_044503858.1	*MiKEA2*	LOC123224297	8	21	1,208	130,774	4.92	0.093	105.38	Plasma membrane
**19**	XP_044464762.1	*MiKEA4*	LOC123195181	13	20	573	61,984.8	6.09	0.635	120.75	Plasma membrane
**20**	XP_044472009.1	*MiKEA6*	LOC123200724	17	20	579	62,521.5	5.39	0.659	124.58	Plasma membrane
**21**	XP_044490612.1	*MiKEA3*	LOC123214717	4	19	806	88,152.1	5.47	0.283	109.98	Plasma membrane
**22**	XP_044506989.1	*MiKEA5*	LOC123226529	9	20	577	62,700.8	6.33	0.628	122.43	Plasma membrane
**23**	XP_044502477.1	*MiTPK4*	LOC123223382	8	3	347	43,193.9	8.82	0.26	107	Plasma membrane
**24**	XP_044496794.1	*MiTPK6*	LOC123219111	6	2	426	47,442.5	8.96	0.094	97.65	Plasma membrane
**25**	XP_044464971.1	*MiTPK1.1*	LOC123195341	1	3	353	39,058.5	5.74	0.265	111.53	Plasma membrane
**26**	XP_044473453.1	*MiTPK1*	LOC123201916	18	5	352	39,220.9	8.31	0.129	99.94	Plasma membrane
**27**	XP_044503723.1	*MiTPK5*	LOC123224194	8	3	341	43,864.1	6.1	0.136	105.96	Plasma membrane
**28**	XP_044462393.1	*MiTPK6.1*	LOC123193455	12	3	388	46,894.7	8.94	0.154	98.11	Plasma membrane
**29**	XP_044496428.1	*MiTPK1.2*	LOC123218838	6	2	340	38,379.9	8.4	0.264	107.53	Plasma membrane
**30**	XP_044506365.1	*MiSKOR1*	LOC123226010	9	13	795	94,330.7	6.07	−0.058	−0.058	Plasma membrane
**31**	XP_044464305.1	*MiAKT1*	LOC123194882	13	11	892	100,795	7.33	−0.156	94.43	Plasma membrane
**32**	XP_044464698.1	*MiAKT6*	LOC123195143	13	12	866	97,411.9	7.86	−0.105	95.61	Plasma membrane
**33**	XP_044471608.1	*MiAKT1.1*	LOC123200477	17	10	873	98,247.9	8.18	−0.091	96.52	Plasma membrane
**34**	XP_044464837.1	*MiKAT3*	LOC123195242	13	13	622	71,509.6	7.36	−0.084	97.62	Plasma membrane
**35**	XP_044490239.1	*MiAKT2*	LOC123214504	4	11	838	95,849.4	6.49	−0.162	95.27	Plasma membrane
**36**	XP_044461595.1	*MiKAT2*	LOC123192942	12	10	823	90,520.2	6.23	−0.238	88.61	Plasma membrane
**37**	XP_044496295.1	*MiKAT2.1*	LOC123218753	6	11	786	89,417.4	6.44	−0.167	90.77	Plasma membrane

### 3.2 Phylogenetic analysis of K^+^ transport-related proteins in *M. indica*

**Figure 1:**
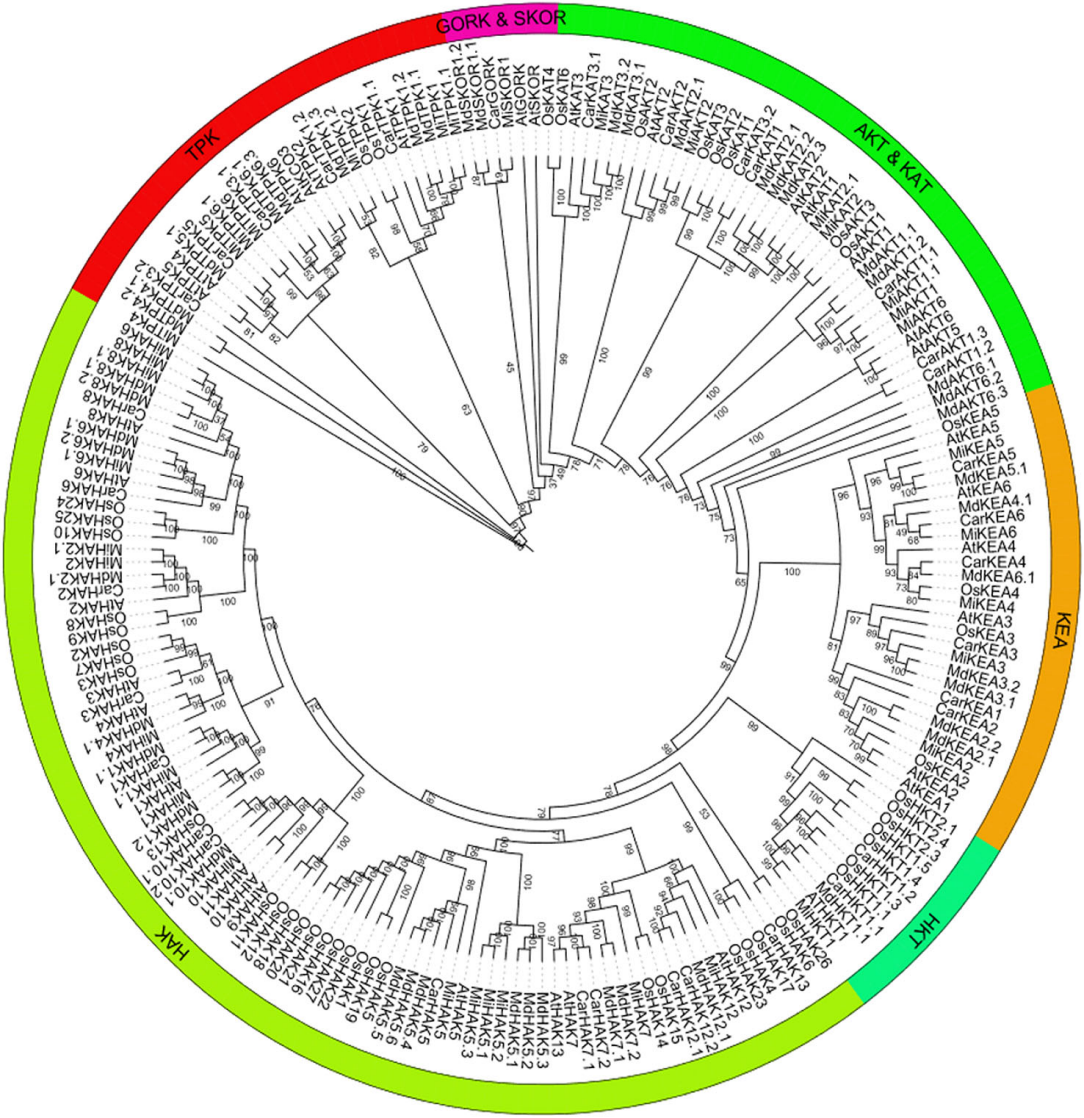
Phylogenetics analysis of K^+^ transporting proteins in *Mangifera indica* (Mi), *Oryza sativa* (Os), *Arabidopsis thaliana* (At), *Cicer arietinum* (Car), and *Malus domestica* (Md). Tree was generated by MEGA-7, using 1,000 bootstrap values with the neighbor-joining method.

To predict the functional properties as well as phylogenetic relationships of the K^+^ transport-related genes, using protein sequences of PTGs of *O. sativa*, *A. thaliana*, *C. arietinum*, *M. domestica*, and *M. indica*, a phylogenetic tree was constructed. The members of the tree were divided into five groups: i) HAK, ii) HKT, iii) KEA, iv) TPK, and v) Shakers (AKT and KAT, and GORK and SKOR). HAK, HKT, and KEA are transporters, and TPK and Shakers are Channels. In phylogenetic analysis, some orthologous pairs (*MiHAK1*/*MdHAK1.2* and *MdHAK6.2*/*MiHAK6.1*), co-orthologous groups (*MdHAK1.1*/*CarHAK1*/*MiHAK1.1*, *MdHAK7.1*/*MdHAK7.2*/*MiHAK7*, and *MdHAK5.4*/*CarHAK5*/*MiHAK5*), and paralogous groups (*MiHAK8*/*MiHAK8.1*, *MiHAK2.1*/*MiHAK2*, and *MiHAK5.3*/*MiHAK5.1*/*MiHAK5.2*) of *M. indica* were identified in the HAK family among *A. thaliana*, *O. sativa*, and *M. domestica*. In the HKT family, one orthologous pair was identified in *A. thaliana* (*MiHKT1*/*ATHKT1*). In the KEA family, two orthologous pairs (*MiKEA6*/*CarKEA6* and *MiKEA4*/*OsKEA4*) and two co-orthologous groups (*MdKEA3.1*/*MdKEA3.2*/*MiKEA3* and *MdKEA5.1*/*CarKEA5*/*MiKEA5*) were identified in the genome of *M. indica*. In the AKT and KAT sub-families, paralogous (*MiAKT1*/*MiAKT1.1* and *MiKAT2*/*MiKAT2.1*) and orthologues groups (*AtAKT6*/*MiAKT6*, *MiAKT2*/*MdAKT2.1*, and *MdKAT3.1*/*MdKAT3.2*/*MiKAT3*) were identified in *M. indica*. In the GORK and SKOR sub-families, one orthologous pair (*MiSKOR1*/*CarGORK*) was identified; in the TPK family, some orthologous and co-orthologous groups were identified (*MdTPK4.1*/*MdTPK4.2*/*MiTPK4*, *MiTPK5*/*CarTPK5*, and *MiTPK1.2*/*MdTPK1.3*); some paralogous groups were also identified (*MiTPK6*/*MiTPK6.1* and *MiTPK1.1*/*MiTPK1*) in the *M. indica* genome (**Figure 1**).

### 3.3 Gene structure and motif detection of K^+^ transport-related proteins in *M indica*

**Figure 2:**
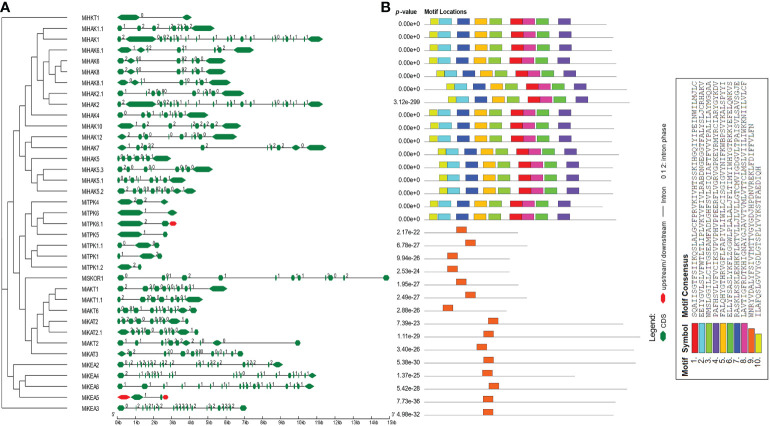
Genomic and proteomic features of PTGs. **(A)** Intron/exon architecture of PTGs in *Mangifera indica*. The green lines indicate introns. The upstream and downstream regions of genes are represented in red color. The exons are represented by green shapes. **(B)** Prediction of conserved motifs PTG proteins from *M. indica*. The conserved motifs are shown in this figure along with their motif details.

To acquire an understanding of the structural properties of PTGs, we explored the intron and exon architecture of these genes in *M. indica*. The results of this analysis demonstrated that the number of exons varies from 2 to 20. In HKTs, there were only two exons. In HAKs, the number of exons ranged from 7 to 12. Similarly, 19–21, 2–5, and 10–13 exons were predicted in KEA, TPK, and Shaker families, respectively (**Figure 2A**). In some regions, CDS are less in concentration and illustrated in green color in the legend. Similarly, conserved motifs were predicted and visualized by using MEME suite. A total of 10 conserved motifs were predicted in all proteins. Among these, motifs 1–8 and 10 were present in all proteins. Motif 9 was detected in only KEAs, TPKs, and Shakers. This motif possesses the characteristic GYGD motif, which acts as a selectivity filter for K^+^ (**Figure 2B**).

### 3.4 Conserved domains and chromosomal distribution PTGs

**Figure 3:**
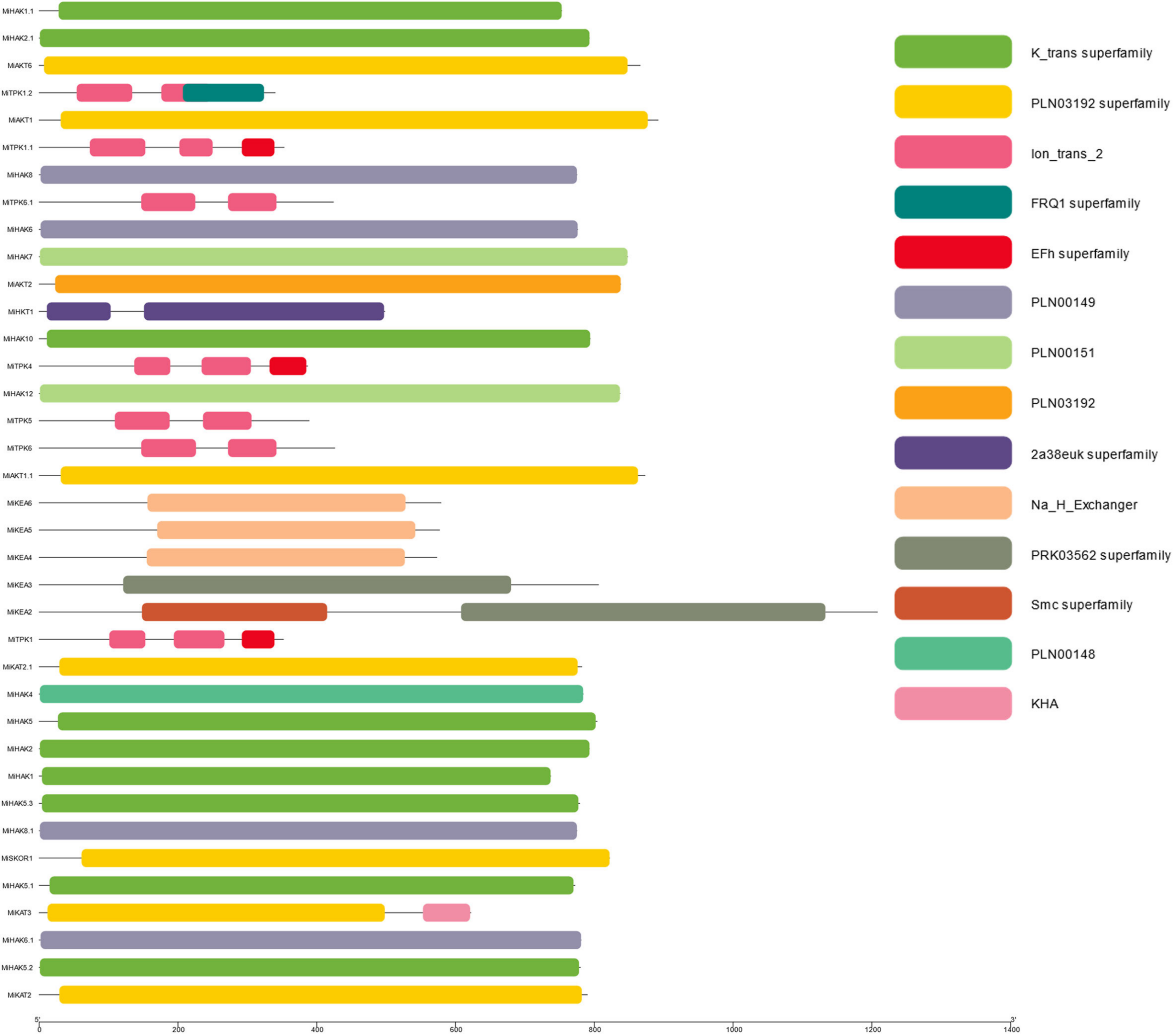
Most conserved domain of K^+^ transporting proteins in *Mangifera indica*.

The conserved domains present in the PTG proteins in *M. indica* were analyzed using NCBI-CDD. After this, further analysis was performed in phases and visualized by using the software Tbtools. All the members of PTGs possess conserved domains like (the K_trans superfamily, ions_trans_2, Na_H_Exchanger, KHA, FRQI superfamily, PLN00149, and PLN00151). Not only the most conserved domains are visualized but less conserved domains are also shown and can be visualized. The least conserved domains are from EFhs Superfamily, which revealed that some proteins function distantly (**Figure 3**).

In *M. indica*, 36 of 37 genes were mapped on 14 (out of 18) chromosomes (**Supplementary Figure 1**). The locus of *MiHAK8.1* gene was not found. As of now, this gene has not been assigned to any linkage groups on scaffolds. Hence, its location is not displayed on the map. There were no PTGs on chromosomes 3, 7, 11, and 16. Chromosome 8 contains the maximum number (5) of genes, while only one gene was present on chromosomes 1, 2, 15, and 18. Almost 40% of members (13 out of 36) were present on chromosomes 8, 12, and 13 collectively (**Supplementary Figure 1**).

### 3.5 *Cis*-regulatory elements analysis

**Figure 4:**
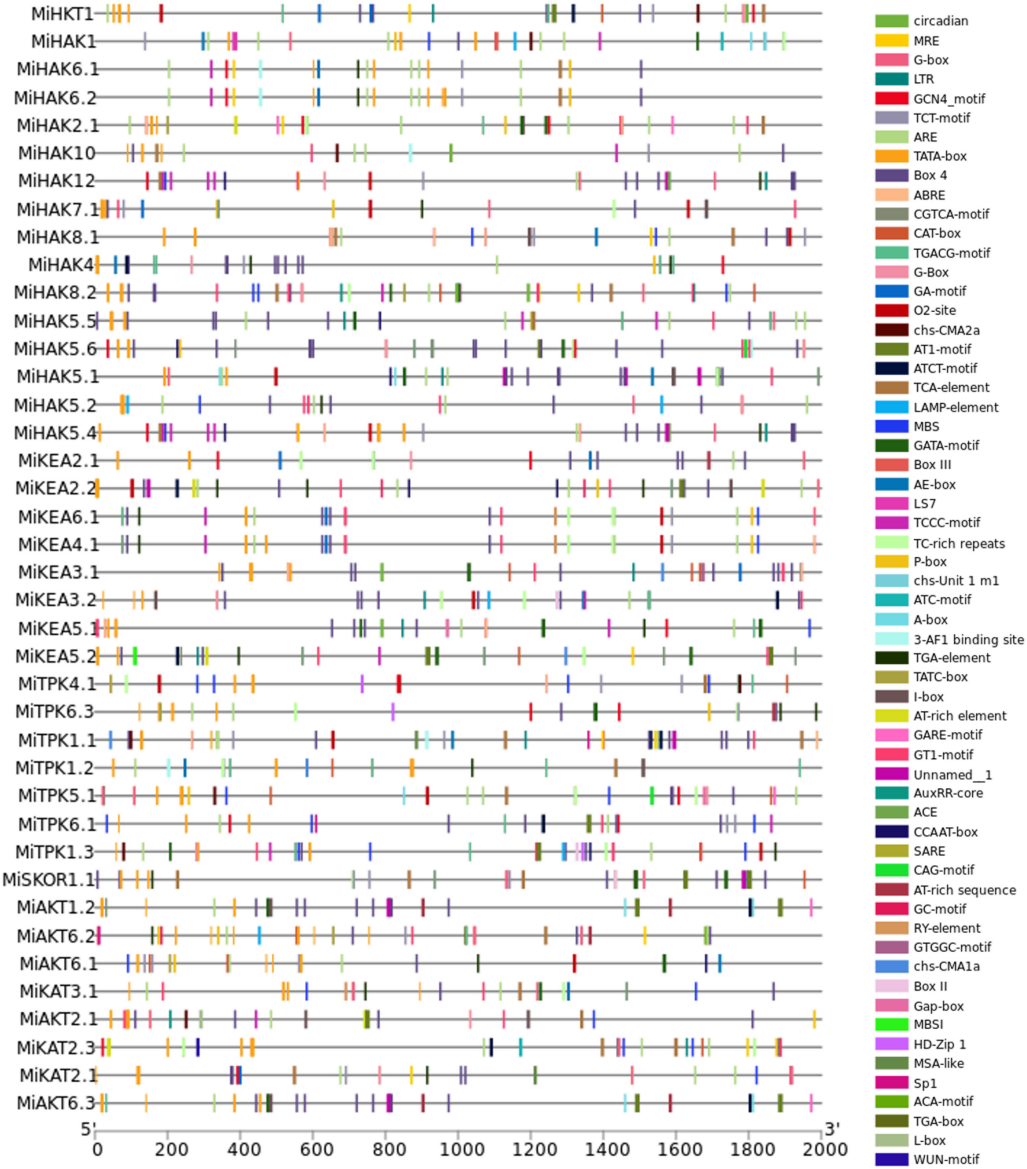
*Cis*-regulatory elements within 2,000-bp promoter region of PTGs.

Gene expression is controlled by *cis*-regulatory sequences, such as enhancers and promoters, which play crucial roles in modulating the development and physiology of an organism. In the current study, we have identified 60 types of *cis*-regulatory elements in the 2,000-bp promoter sequences of 37 PTGs in *M. indica*. The promoter sequence of each gene was found to be rich in these regulatory elements. Most abundant of them all were found to be TATA-Box (TATA-box is capable of defining the direction of transcription and also indicates which DNA strand should be read by the transcriptional machinery), CAAT-box (CAAT-box serves as a marker for the binding site of the transcription factor for RNA), O2-site (assists in regulating two transcription factors, those associated with the metabolism of carbon and amino acids as well as the resistance to abiotic stress), AT-rich element (a replication complex is formed at this specific site and DNA synthesis is initiated), CAT-box (*cis*-acting regulatory element related to meristem expression), ARE (regulatory element essential for anaerobic induction), MRE (MYB binding site involved in light responsiveness), MBS (MYB binding site involved in drought-response), BOX-4 (conserved DNA module involved in light responsiveness), TCA-element (regulatory element involved in salicylic acid responsiveness), ABRE (Abscisic Acid-Responsive Element), and P-box (gibberellin-responsive element); in addition to these, other regulatory elements were also identified, which were not present in the promoter sequence of all the genes and were found to be present in a very small number but could have a potentially significant role in the life of *M. indica* plant, which includes Circadian, MREG-box, LTRGCN4_motif, TCT-motif, CGTCA-motif, TGACG-motif, G-Box, GA-motif, chs-CMA2aAT1-motif, ATCT-motif, LAMP-element, GATA-motif, Box III, AE-box, LS7TCCC-motif, TC-rich repeats, chs-Unit 1, m1ATC-motif, A-box, 3-AF1 binding site, TGA-element, TATC-box, I-box, GARE-motif, GT1-motif, AuxRR-core, ACECCAAT-box, SARECAG-motif, AT-rich sequence, GC-motif, RY-element, GTGGC-motif, chs-CMA1a, Box II, Gap-box, MBSIHD-Zip 1, MSA-like, Sp1, ACA-motif, TGA-box, L-box, and WUN-motif (**Figure 4**).

### 3.6 Expression profiling based on NGS data analysis of drought stress response and fruit development

**Figure 5:**
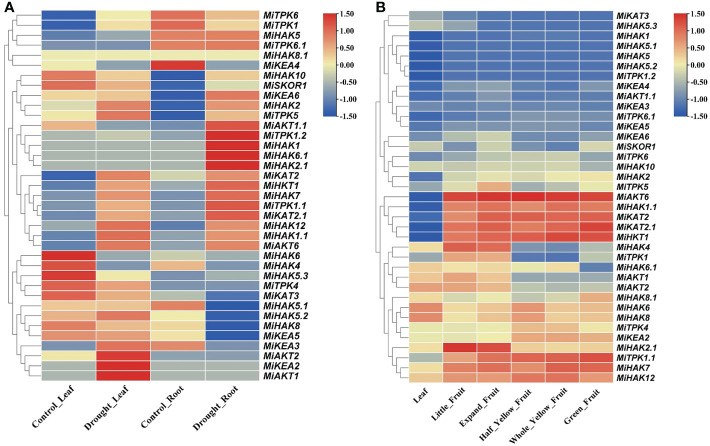
**(A)** Profiles of PTG expression in leaves and roots of *Mangifera indica*. The expression values [log2(FPKM)] are shown by the colored bar. Red denotes high expression, yellow denotes no expression, and blue represents low expression. **(B)** Profiles of PTG expression during fruit development. The expression values [log2(FPKM)] are shown by the colored bar. Red denotes high expression, whereas blue denotes low expression.

To check the gene expression profile of PTGs in *M. indica* leaflets and roots under drought stress, the gene expression data of *M. indica* were downloaded from the NCBI-SRA database (**Figure 5A**). The differentially regulated genes were either upregulated in both leaves and roots (*MiKAT2.1*, *MiHAK2*, *MiTPK5*, *MiHKT1*, *MiHKT7*, *MiTPK1.1*, *MiHAK12*, *MiHAK1.1*, *MiAKT6*, and *MiHKT1*), upregulated in leaves and downregulated in the root (*MiAKT1*, *MiKEA2*, *MiAKT2*, *MiKEA3*, *MiKEA5*, *MiHAK8*, *MiHAK5.2*, *MiHAK5.1*, *MiKAT3*, and *MiTPK4*), and upregulated in root and downregulated in leaves (*MiHAK5*, *MiTPK6.1*, *MiAKT1.1*, *MiTPK1.2*, *MiHAK1*, *MiHAK6.1*, and *MiHAK2.1*), while the rest of the genes were non-responsive (**Figure 5A**). Interestingly, *MiTPK1.2*, *MiHAK1*, *MiHAK2.1*, *HAK6.1*, and *MiAKT1.1* were most upregulated in roots, and *MiKEA2*, *MiAKT2*, and *MiAKT1* were upregulated in leaves.

RNA-seq data were also used to analyze the gene expression profile of PTGs during the five stages of fruit ripening in *M. indica*. SRA data for the bioProject (PRJNA797728) were retrieved from the NCBI-SRA database. Gene expression profiles were divided into two groups, i.e., those genes that were upregulated in five different stages of fruit ripening (*MiAKT6*, *MiHAK1.1*, *MiKAT2*, *MiKAT2.1*, *MiHKT1*, *MiTPK1.1*, *MiHAK7*, and *MiHAK12*) and those genes that were highly downregulated in leaves and fruit ripening according to FPKM values (*MiHAK1*, *MiHAK5.1*, *MiHAK5*, *MiHAK5.2*, and *MiTPK1.2*), and the rest of the genes showed non-responsive or non-significant variations of expression (**Figure 5B**).

### 3.7 Relative expression profiling of PTGs using real-time RT-qPCR

**Figure 6:**
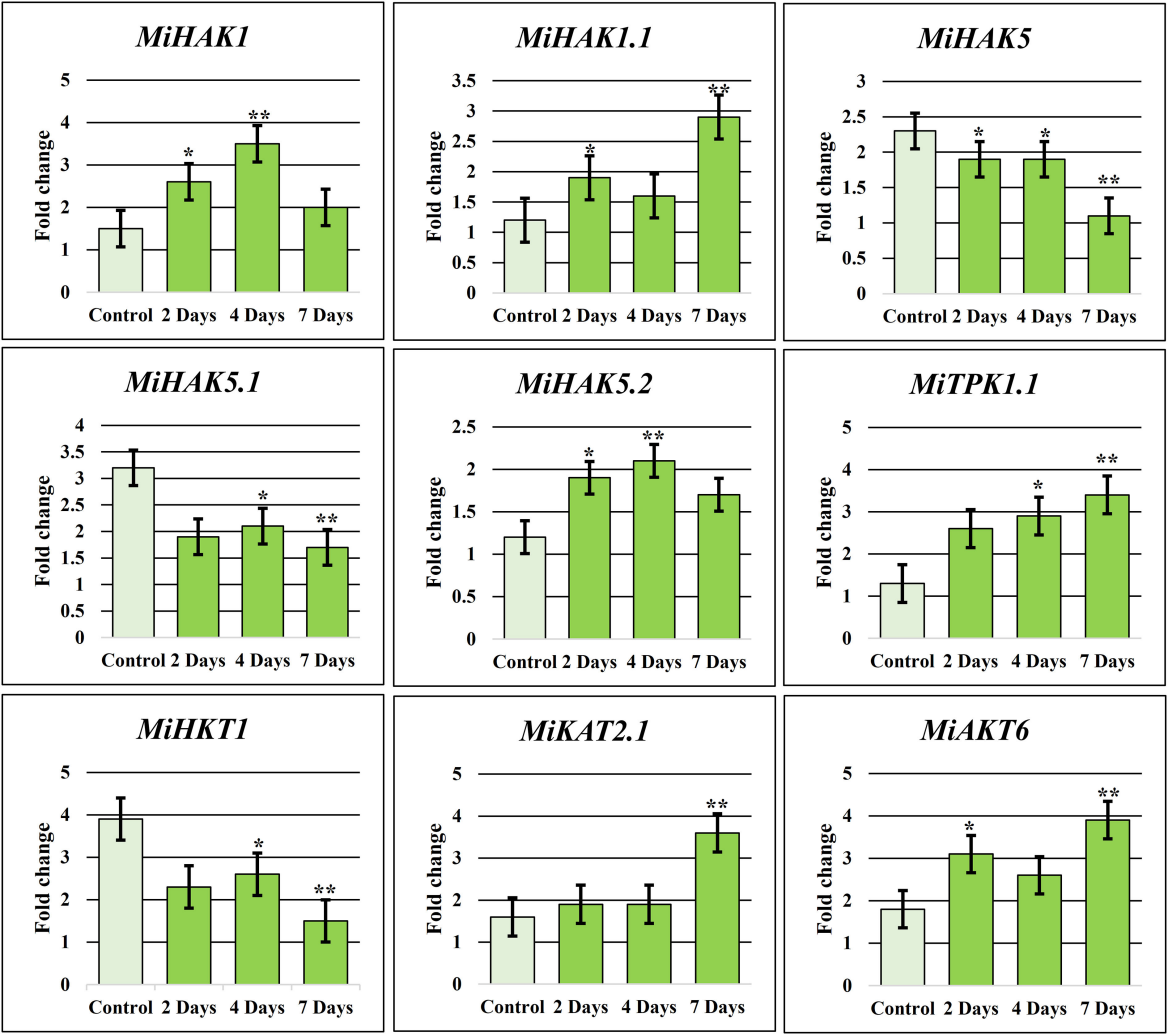
K^+^ transporting gene family relative qRT-PCR in response to drought. To produce an objective average value, the experiment was triplicated. In untreated plants, each gene had a default expression value of 1. Bars have been placed on each column to illustrate the standard error. *denotes significant differences between environmental stressors and the control (p 0.05), whereas **denotes extremely significant differences (p 0.001).

Real-time amplification (qRT-PCR) was performed to confirm the expression of nine selected PTGs in *M. indica* leaves. These genes were selected based on their expression values in drought stress response (**Figure 5A**) and higher expression during fruit development (**Figure 5B**). In response to drought stress, nine genes, including *MiAKT6*, *MiHAK1.1*, *MiKAT2.1*, *MiHKT1*, *MiTPK1.1*, *MiHAK1*, *MiHAK5.1*, *MiHAK5*, and *MiHAK5.2*, were selected for qPCR-based quantification for 7 days, and samples were taken at the second, fourth, and seventh days. Under drought stress, *MiHAK1* was regulated differently. Although according to RNA-seq analysis *MiHAK1* was downregulated in response to drought stress, it was found to be upregulated up to 3.5-fold in qPCR. Similarly, *MiHAK1.1*, *MiHAK5.2*, *MiTPK1.1*, *MiKAT2.1*, and *MiAKT6* were significantly upregulated after drought stress. On the contrary, *MiHAK5*, *MiHAK5.1*, and *MiHKT1* were significantly downregulated (**Figure 6**). There was a significant correlation between the results of qPCR and RNA-seq data. However, some variations were also observed. Though similar plant growth and stress conditions were applied, such variations are potentially associated with sampling time in a day, plant age, or even genotypic differences.

## 4 Discussion

### 4.1 Potassium transport system is highly conserved in *M indica* and other plants

The current study identified 37 potential genes of the K^+^ transport system in *M. indica* genome. The number of PTGs is more or less similar in major plant groups represented by *V. vinifera*, *Glycine max*, *Triticum aestivum*, *Vigna radiata*, *C. cajan*, and *A. thaliana* (Cuéllar et al., 2010; Isayenkov et al., 2011; Chen et al., 2017; Azeem et al., 2018; Azeem et al., 2021a; Azeem et al., 2021b; Ge et al., 2020; Siddique et al., 2021; Cai et al., 2021). It not only indicates the evolutionary conservation of PTGs in these plants but also predicts the existence of similar genetic mechanisms for K^+^ in other fruit trees. A total of 202 PTG protein sequences from five species—*A. thaliana*, *M. indica*, *M. domestica*, *O. sativa*, and *C. arietinum*—were divided into six groups according to phylogenetic analyses (HAK, HKT, KEA, AKT and KAT, GORK and SKOR, and TPK). In contrast, the phylogenetic relationship analysis showed that *PTG*s were more closely related to *MdPTG*s than *AtPTG*s. This outcome confirmed the finding that apple and mango also showed a closer association than *Arabidopsis*. It is also supported by the fact that various sequence features were common in *M. indica* and other species. For example, the transmembrane domains of MiHKT1.1 and AtHKT1;1 are eight. Similarly, the presence of the consensus motif GVVYGDLGTSPLY is a characteristic feature of HAK transporters (Cheng et al., 2018). This motif was present with a minor modification as GVVYGDLG(I/T)SPLY. This variation of a motif is also present in HAK proteins in *A. thaliana* and *Camellia sinensis* (Yang et al., 2020). KEA members of both *M. indica* and *A. thaliana* species showed conservation of “FLLFxxGLE and GEFAFVxxxxA” motifs. Previously, it has been established that a divergence in the amino acid sequences of proteins is related to the functional divergence of proteins and vice versa (Sangar et al., 2007). Among K^+^ channels, the presence of ANK, Ion_trans_2, and KHA domains in the Shaker proteins, similar to *Arabidopsis* K^+^ channels, suggests their functional similarities (Keisham et al., 2018). Likewise, the occurrence of the significantly conserved residues, i.e., GYGD and RSXpS/pTXP, in MiTPKs indicates potential functional similarities in *M. indica* and other plants (Ge et al., 2020; Siddique et al., 2021).

### 4.2 Potassium transport system is involved in drought stress response in *M. indica*

As one of the most abundant cations in plant cells, K^+^ performs a dominant role in various biological processes of plants (Ragel et al., 2019). The influx/efflux of K^+^ (mediated by channel and transporter proteins) regulates its concentration in the plant body. K^+^ channels and transporters are important contributors to plant growth and development (Sharma et al., 2013). In the current study, members of HAK, HKT, TPK, and Shaker families were differentially regulated by drought stress. These results were more or less in agreement with RNA-seq and qPCR analyses. Previously, the role of potassium channels and transporters has been documented in the drought stress response of various plants (Ahmad et al., 2016a; Ahmad et al., 2016b; Chen et al., 2017; Hassan et al., 2017; Cai et al., 2019; Qi et al., 2019; Singh et al., 2021). The overexpression of the *OsAKT1* and *OsHAK1* significantly affects potassium nutrition and drought stress tolerance of rice (Ahmad et al., 2016a; Chen et al., 2017). Our results also proposed similar roles of K^+^ transporters. Overexpression of the potassium channel TPKb in small vacuoles confers osmotic and drought tolerance to rice (Ahmad et al., 2016b). Taken together, these findings suggest a potential role of PTGs in the stress response of the *M. indica* tree.

### 4.3 *M. indica* fruit development involves differential regulation of PTGs

The essential nutrients (N, P, and K) promote an increase in biomass yield by improving root growth, activating cellular enzymes, ameliorating photosynthesis, conserving energy, and positively affecting other fundamental processes in the plant body. Potassium sources can influence the K^+^ release and fruit yield of *M. indica* (Wang et al., 2022). It emphasizes the importance of potassium uptake and transport system from root to leaves and fruit. The current study reports significant differential regulation of K^+^ transport during five stages of mango fruit development. Previously, it is reported that supplemental foliar applications of K^+^ can improve fruit quality in *Cucumis melo* (Lester et al., 2005), *Solanum lycopersicum* (Liu et al., 2021), *C. sinensis* (Wu et al., 2021), and *M. indica* (Sarker and Rahim, 2013). The findings of the current study complement the potential role of PTGs in improving mango fruit growth, development, and quality.

## Conclusion

In the current study, the K^+^ transport system was characterized in *M. indica*. There are 37 potential members of PTGs in this plant. Among these, 22 genes can be classified as K^+^ transporters, and the remaining 15 genes are K^+^ channels. Moreover, the analysis performed including conserved domain and TM domain prediction, motif analysis, phylogenetic analysis, and genetic structure display, and *cis*-regulatory elements predicted in the promoter regions revealed the close relation of PTGs in several plant species. Differentially expressed genes *MiHKT7*, *MiHAK5.1*, *MiHAK5*, *MiTPK1.1*, and *MiHAK12* were responsive to drought stress. Moreover, *MiHKT7*, *MiHAK5.1*, *MiHAK5*, *MiTPK1.1*, and *MiHAK12* genes were also differentially expressed during different growth stages of mango fruit development. To gain depth of information about the K^+^ transport system, these genes can be selected for further genomic and biotechnological study and to improve stress response and fruit quality in mango.

## Data availability statement

The datasets presented in this study can be found in online repositories. The names of the repository/repositories and accession number(s) can be found in the article/**Supplementary Material**.

## Author contributions

Conceptualization: FA. Data curation: TL and MW. Formal analysis: TL and AR. Funding acquisition: FA and TL. Investigation: TL and MR. Methodology, MW and AR. Project administration: FA. Resources: FA. Software: MW and AR. Supervision: FA. Validation: FA, TL, and HM. Visualization: MW. Writing—original draft: TL. Writing—review and editing: FA and HM. All authors contributed to the article and approved the submitted version.

## Supplementary material

Supplementary Figure 1 Distribution of PTGs on *M. indica* on chromosomes. The 37 PTGs were mapped onto all chromosomes except 3, 7, 11, and 16. Each gene is represented by a specific color.
