# Supplementary material for: Computational analysis and expression profiling of potassium transport-related gene families in mango (Mangifera indica) indicate their role in stress response and fruit development
Source: Front Plant Sci. 2023 Jan 23;13:1102201. doi: 10.3389/fpls.2022.1102201 (PMC9899903; doi:10.3389/fpls.2022.1102201)
Supplement: Supplementary Figure 1 — Distribution of PTGs on M. indica on chromosomes. The 37 PTGs were mapped onto all chromosomes except 3, 7, 11, and 16. Each gene is represented by a specific color. [file DataSheet_1.docx]

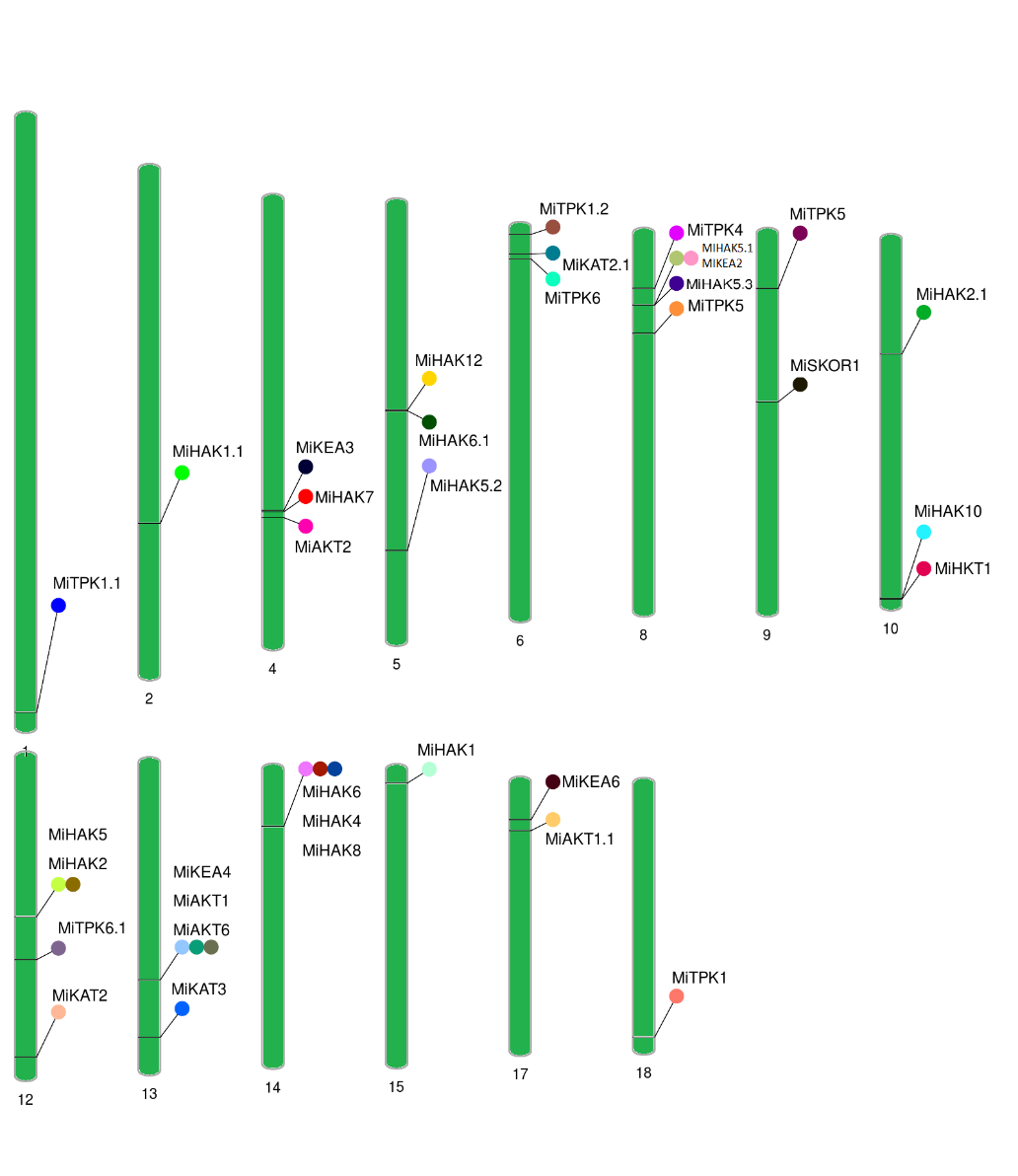


Supplementary Figure 1: Distribution of PTGs on *M. indica* on chromosomes. The 37 PTGs were mapped onto all chromosomes except 3, 7, 11, and 16. Each gene is represented by a specific color.
